# Advances in the potential roles of Cullin-RING ligases in regulating autoimmune diseases

**DOI:** 10.3389/fimmu.2023.1125224

**Published:** 2023-03-17

**Authors:** Xiaoying Zhang, Yu’e Liu, Tong Zhang, Yuying Tan, Xiangpeng Dai, Yong-Guang Yang, Xiaoling Zhang

**Affiliations:** ^1^ Key Laboratory of Organ Regeneration and Transplantation of Ministry of Education, First Hospital, Jilin University, Changchun, China; ^2^ National-Local Joint Engineering Laboratory of Animal Models for Human Disease, First Hospital, Jilin University, Changchun, China; ^3^ Tongji University Cancer Center, Shanghai Tenth People’s Hospital of Tongji University, School of Medicine, Tongji University, Shanghai, China; ^4^ International Center of Future Science, Jilin University, Changchun, China

**Keywords:** Cullin-RING ligases, autoimmune diseases, E3 ligases, ubiquitination, inflammation

## Abstract

Cullin-RING ligases (CRLs) are the largest class of E3 ubiquitin ligases regulating the stability and subsequent activity of a large number of important proteins responsible for the development and progression of various diseases, including autoimmune diseases (AIDs). However, the detailed mechanisms of the pathogenesis of AIDs are complicated and involve multiple signaling pathways. An in-depth understanding of the underlying regulatory mechanisms of the initiation and progression of AIDs will aid in the development of effective therapeutic strategies. CRLs play critical roles in regulating AIDs, partially by affecting the key inflammation-associated pathways such as NF-κB, JAK/STAT, and TGF-β. In this review, we summarize and discuss the potential roles of CRLs in the inflammatory signaling pathways and pathogenesis of AIDs. Furthermore, advances in the development of novel therapeutic strategies for AIDs through targeting CRLs are also highlighted.

## 1 Introduction

Autoimmune diseases (AIDs) refer to autoantibody and autoreactive immune cells attacking self-tissues and inducing severe inflammatory reactions, which damage a variety of host organs including the skin, kidneys, joints, bowel, and the nervous system, among others (1, 2). AIDs can be classified as either organ-specific or systemic AIDs based on whether a single organ or a system, respectively, is damaged. Organ-specific AIDs include Hashimoto’s thyroiditis (HT), pemphigus, insulin-dependent diabetes mellitus (IDDM), and ulcerative colitis (UC). Systemic AIDs include multiple sclerosis (MS), systemic lupus erythematosus (SLE), and rheumatoid arthritis (RA) (3, 4). Environmental risk factors and genome instability are the common leading causes of AIDs (5). The immune system can recognize self from non-self in physiological conditions. However, in certain conditions, environmental triggers such as chemical toxicants, pollution, infection, or intrinsic genome changes can break the immune tolerance and generate excessive autoantibody and autoreactive lymphocytes, which will attack the host tissues and result in the onset of AIDs (3, 6, 7). However, the detailed mechanisms of the pathogenesis of AIDs remain largely unknown and need further in-depth investigation.

In recent years, a lot of studies have shown that the posttranslational modification (PTM) of proteins plays a pivotal role in the occurrence and progression of AIDs (8–10). Ubiquitination, one of the important PTM types, regulates the protein stability, activity, subcellular localization, and interactions in key signaling pathways, subsequently influencing the cell cycle, proliferation, apoptosis, autophagy, and inflammation (10–13). Dysregulation of the ubiquitination of critical proteins could induce excessive immune activation and AIDs (14, 15). The modification of proteins by ubiquitin is a reversible process controlled by ubiquitination and de-ubiquitination. De-ubiquitination modulates the ubiquitin removed from substrates, which is regulated by deubiquitinating enzymes (DUBs). On the other hand, ubiquitination is catalyzed by a cascade of reactions involving three types of key enzymes: ubiquitin-activating enzymes (E1), ubiquitin-conjugating enzymes (E2), and ubiquitin ligases (E3) (11, 13). Ubiquitin is activated in an ATP-dependent manner by E1s and forms a thioester between the ubiquitin C-terminal carboxyl and the E1 active site sulfhydryl of cysteine. Subsequently, ubiquitin transfers from the E1 to the E2 active site cysteine and forms another thioester complex through the catalysis of E2s. In the final steps, E3s catalyze the ubiquitin from E2s to the lysine ϵ-amino group of substrates *via* isopeptide bonds (11, 16). The E3s play a pivotal role in the ubiquitination cascade because they are responsible for substrate specificity.

There are more than 600 E3s in humans, which comprise three families: the really interesting new gene (RING) family, the homology to E6AP C-terminus (HECT) family, and the RING-between-RING (RBR) family (16). The HECT and RBR E3s mediate the transfer of ubiquitin from E2 to the cysteine of the E3 active site and then transfer the ubiquitin from E3 to specific substrates. However, the RING E3s lack the cysteine of active sites and directly transfer the ubiquitin from the E2 ubiquitin intermedia to the substrates (17, 18). Cullin-RING ligases (CRLs), the largest subfamily of the RING E3 ligases, consist of 300 members distributed into different subclasses according to the Cullins. In humans, there are eight Cullin proteins—Cul1, Cul2, Cul3, Cul4A, Cul4B, Cul5, Cul7, and Cul9—that are organizers of the CRL complex (19). CRLs are responsible for the ubiquitination and subsequent degradation of approximately 20% of the proteins regulated by the ubiquitination proteasome system (UPS) in mammalian cells (20, 21). The CRLs play a crucial part in regulating autoimmunity and homeostasis in physiological and pathological conditions (22–24). Previous studies suggested that the F-box and WD repeat domain-containing 7 (FBW7), as a substrate receptor (SR) of SKP1–Cul1–F-box (SCF), was predominantly upregulated in the colon tissues of patients with inflammatory bowel disease (IBD) and was correlated with its severity (25, 26). In addition, the expression of DCAF2, which is a SR of CRL4, was significantly suppressed in biopsies from patients with psoriasis. DACF2 deficiency mediated the activation of the nuclear factor kappa B (NF-κB) signaling pathway and accelerated the severity of psoriasis (23). In this review, we summarize and discuss the potential roles of CRLs in AIDs and provide new insights for AIDs therapy *via* targeting CRLs.

## 2 Cullin-RING E3 ligases: Composition and mechanism

### 2.1 Composition and catalytic mechanism of CRLs

The multi-subunit CRL complex consists of the RBX1 or the RBX2 RING protein, the scaffold Cullin protein, and the adaptor and SR unit. The N-terminal domain of Cullins binds to the SRs with or without the adaptor protein SKP1 or Elongins B/C. The C-terminal domain of Cullins connects with the RING subunit (RBX1 for Cul1–Cul4 and RBX2 for Cul5) (19, 27, 28). The RING domains are responsible for the binding to the E2s and mediates the transfer of ubiquitin, while the SRs recognize and recruit specific substrates. The Cullins, as scaffold proteins, are essential for the assembly of the whole E3 complex and its functions (18, 19, 27).

### 2.2 The subfamily of CRLs and their distinct functions

The CRLs are divided into eight subfamilies according to the eight types of Cullins (Cul1, Cul2, Cul3, Cul4A, Cul4B, Cul5, Cul7, and Cul9). Importantly, each CRL has a distinct composition and structure. The first identified CRL is SCF E3 ligase, in which SKP1, as the adaptor protein, mediates the linkage between Cul1 and the F-box SR protein (27). There are 69 F-box proteins that determine the substrate diversity and specificity in humans. SCF ligases catalyze the mono- or poly-ubiquitination of substrates and affect various cellular processes such as the cell cycle, DNA damage and repair, and other signaling pathways (29). The adaptor proteins for Cul2- or Cul5-based RING E3 ubiquitin ligases are Elongins B and C, which mediate the linkage between the N-terminal of Cullin and the BC-box SRs (30). The first identified substrate of CRL2 is hypoxia-inducible factor 1α (HIF-1α), which regulates the hypoxia response (28, 31). CRL5 has similar components of adaptors and SRs to CRL2. However, the RING unit of CRL5 is RBX2. CRL5 is mainly responsible for signaling transduction, virus infection, and tumorigenesis (32, 33). For CRL3, the SRs directly interact with the Cul3 scaffold protein without the adaptor proteins Skp1 or Elongins B/C. The SRs of CRL3 are usually BTB/POZ (Broad-complex, Tramtrack, and Bric-a-brac/pox virus and zinc finger) domain proteins that share a fold with Skp1. The MATH and Kelch domains of the CRL3 SRs are generally associated with the BTB domain, which is responsible for substrate recognition and recruitment. CRL3 plays a crucial role in regulating the oxidative stress response, cellular homeostasis, tumorigenesis, and progression (28, 34). CRL4 includes two homologous Cullin proteins, i.e., Cul4A and Cul4B, that share the same adaptor protein and the SR proteins including damage-specific DNA binding protein 1 (DDB1) and DDB1/CUL4-associated factor (DCAF). CRL4s have great impact on the disorder of the nervous system and oncogenesis (35). CRL7 and CRL9 are two novel CRLs reported in recent years. Only two F-box proteins Fbxw8 and Fbxw11 have been identified as the SRs of CRL7. Moreover, Cul7, as a scaffold protein, interacts with the RBX1 RING protein and the Skp1 adaptor protein. Importantly, CRL7 could mediate proteolytic and non-proteolytic ubiquitination. There are approximately 10 substrates in total in the involvement of CRL7 in the regulation of cell proliferation, apoptosis, and DNA damage repair (36, 37). The exact components of CRL9 are largely unknown. CRL9 has been reported to mediate the ubiquitination and degradation of survivin and to maintain genome integrity. CRL9 also acts as an activator of p53 to inhibit cell proliferation and promote DNA damage repair. Therefore, CRL9 has been considered a tumor suppressor due to its function in the regulation of p53 and survivin (38–40).

### 2.3 Regulation of CRLs *via* NEDDylation, substrate adaptor exchange, and phosphorylation

The process of protein ubiquitination and the subsequent proteolytic or non-proteolytic functions that are controlled by CRLs can also be regulated by several mechanisms. The protein NEDD8 can covalently attach to a lysine moiety located in the WHB (winged-helix) domain of Cullins and enhance the activity of CRLs (41). Similar to ubiquitination, NEDDylation is a reversible process. It is catalyzed by an enzymatic cascade with the E1 NEDD8-activating enzyme (NAE), E2 NEDD8-conjugating enzyme, and E3 NEDD8 ligase. The COP9 signalosome (CSN) mediates the deNEDDylation of Cullins. The processes of NEDDylation/deNEDDylation regulate the activation of CRLs and influence the fate of the substrates of CRLs (20, 42).

Previous biochemical studies suggested that Cullin-associated and NEDDylation-dissociated protein 1 (CAND1) is a negative regulatory factor of SCF. CAND1 binds to the unNEDDylated Cul1 and inhibits the assembly of the SCF complex by blocking the combination of the adaptor protein Skp1 with Cul1 (43, 44). However, recent genetic studies have identified that CAND1 also promotes the activation of SCF; moreover, the CAND1 mutant showed a reduction of the SCF activity in *Arabidopsis* (45). A growing body of later studies demonstrated that CAND1, as an exchange factor, mediates the dynamic exchange of F-box–Skp1 substrate adaptors and regulates the substrate specificity of SCF (46–48).

The crosstalk between the phosphorylation and ubiquitination mediated by CRLs is prevalent in eukaryotic cells. The phosphorylation of substrates often inhibits or promotes their recognition and interaction with CRLs. It is well established that the SR of SCF, FBW7, recognizes and interacts with its substrates after the phosphorylation of special amino acids in their degron (49). In addition, as the SR of CRL3, the speckle-type POZ protein (SPOP) often recognizes the substrates with conserved Ser/Thr-rich motifs. In most cases, phosphorylation in the motif is essential for the interaction between substrates and SPOP (50). However, a previous study indicated that casein kinase 1 (CK1) mediated the phosphorylation of Ci at the Ser/Thr-rich degrons and inhibited the interaction between Ci and MATH and BTB domain-containing protein (HIB/SPOP), which is a SR of CRL3 (51). On the other hand, the phosphorylation of the adaptor or SR of CRLs also influences the substrates’ binding to CRLs and the subsequent ubiquitination and degradation (50). Targeting the CRL pathways might be a promising strategy for the treatment of related diseases through regulating the abundance of the key proteins in human diseases. Identification of more detailed mechanisms of the regulation of CRLs will help in the development of CRL-targeted therapy.

## 3 Critical signaling pathways of immunity and inflammation regulated by CRLs

### 3.1 NF-κB signaling pathway

NF-κB, as a transcription factor, regulates the expression of a series of genes involved in multiple cellular processes including inflammation, autoimmunity, and cell survival (52, 53). The NF-κB signaling pathway consists of canonical and non-canonical pathways. The transcription factor complexes assembled by p65 (RelA) and p50 can translocate into the nucleus and promote the transcription of a set of target genes, which leads to the activation of the canonical NK-κB signaling pathway. The p65/p50 complex is maintained in the cytoplasm by IkappaB (IκB), an inhibitor of the canonical NF-κB pathway. IκB can be phosphorylated by IκB kinase (IKK) and subsequently degraded by the 26S proteasome to release the p65/p50 complex from cytoplasmic retention (52, 54). RelB/p52 heterodimers are responsible for the transcriptional activation of the target genes in the non-canonical NF-κB signaling pathway. p100, the precursor of p52, acts as an inhibitor that blocks the translocation of RelB into the nucleus. The proteolysis of p100 results in the production of p52 and constitutes the RelB/p52 complex, which is important for the activation of the non-canonical NF-κB pathway (54, 55).

**Figure 1:**
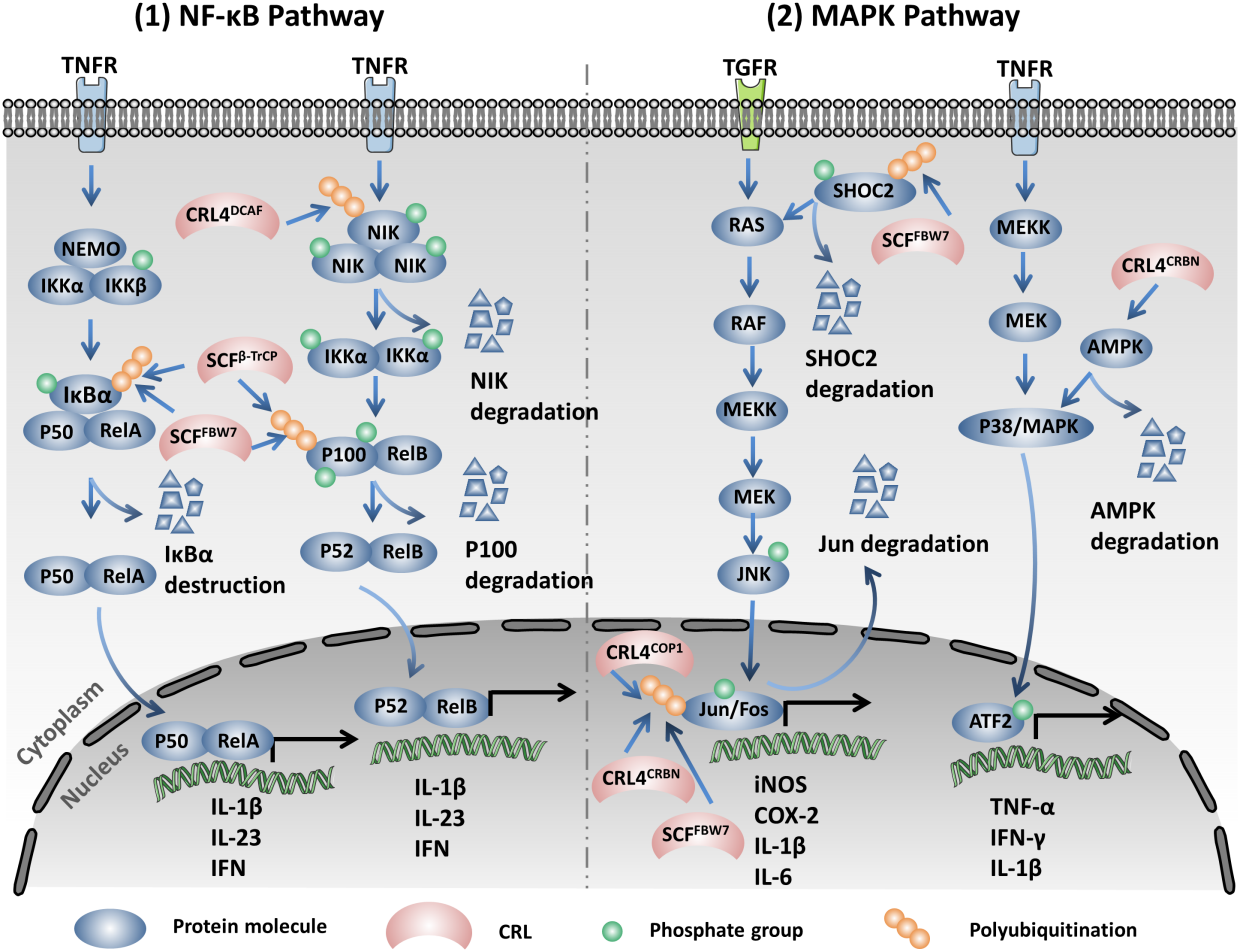
Cullin-RING ligases (CRLs) regulate the expression of multiple pro-inflammatory cytokines through modulating the nuclear factor kappa B (NF-κB) and mitogen-activated protein kinase (MAPK) signaling pathways. *Left*: SCF^β-TrCP^ mediates the degradation of the NF-κB inhibitor IκB after phosphorylation by IκB kinase (IKK). CRL4^DCAF2^ mediates the poly-ubiquitination and destruction of the NF-κB-inducing kinase (NIK). SCF^β-TrCP^ modifies p100 at the C-terminal domain and induces degradation, generating the p52 mature subunit. SCF^FBW7^ recognizes and destroys the IκB and p100 in the ubiquitination proteasome system (UPS). These CRLs modulate the NF-κB pathway through regulating the stability of the key components. *Right*: SCFFBW7 mediates the ubiquitination and proteolysis of SHOC2 after being phosphorylated by MAPK at Thr507 and blocks the RAS-MAPK pathway. CRL4^CRBN^ promotes the K48 linkage ubiquitination and the subsequent degradation of c-Jun and restrains the activity of the AP-1 complex. COP1, another Cul4-based E3 ubiquitin ligase, also regulates the protein stability of c-Jun. SCF^FBW7^ mediates the ubiquitination and destruction of c-Jun by proteasome. CRL4^CRBN^ promotes a non-K48 linkage ubiquitination and the degradation of AMPKa.

CRLs regulate the activity of NF-κB both in the canonical and non-canonical signaling pathways (**Figure 1**, left). SCF^β-TrCP^ mediates the degradation of the NF-κB inhibitor IκB after its phosphorylation by IKK and releases the p65/p50 complex, which translocates into the nucleus and performs transcriptional activity in the NF-κB canonical pathway (56, 57). A recent study has reported that SCF^FBW7^ promoted the ubiquitination and proteolysis of IκB. The upregulation of FBW7 promoted the activation of the NF-κB pathway through the negative regulation of IκB and accelerated the intestinal inflammation in intestinal epithelial cells (IECs) (25). In addition, SCF^β-TrCP^ modifies p100 at the C-terminal domain, induces degradation, and generates the p52 mature subunit, constitutes the RelB/p52 complex then translocating into the nucleus and activating NF-κB in the non-canonical pathway (58, 59). SCF^FBW7^, another SCF E3 ligase, could recognize p100 based on the conserved degron in a GSK3-dependent manner. SCF^FBW7^ recognizes and destroys the p100 in the UPS after being phosphorylated by GSK3 at the Ser 707 and 711 sites, which subsequently contributes to the activation of the non-canonical NF-κB pathway (60–62). The Regulator of Cullins 1 (ROC1) mediates p-IκBα ubiquitination and the subsequent degradation induces the nuclear translocation of the P65 subunit and NF κB activation in bladder cancer. CRL5^SPSB1^ negatively regulates the activity of NF-κB through an undefined mechanism (63). Early studies have suggested that the NF-κB-inducing kinase (NIK) can be subjected to proteolysis in a TRAF3-dependent manner. TRAF3 mediates the ubiquitination and degradation of NIK by recruiting it to the cIAP1–cIAP2–TRAF2 ubiquitin ligase complex (64, 65). However, recent research has indicated that CRL4^DCAF2^ mediates the poly-ubiquitination and destruction of NIK in a TRAF3 pathway-independent manner. It was also found that CRL4^DCAF2^ inhibits the non-canonical activity of NF-κB in a NIK-dependent manner and ultimately reduces the production of interleukin 23 (IL-23), which can be a potential therapeutic target for psoriasis (23).

### 3.2 MAPK signaling pathway

It is well known that the mitogen-activated protein kinase (MAPK) signaling pathway plays a central role in cell proliferation, apoptosis, and inflammation. Three main subgroups namely, extracellular signal-regulated kinase (ERK), c-Jun N-terminal kinase (JNK), and p38 build up the MAPK signaling pathway. The ERK pathway mainly regulates cell proliferation and differentiation. However, the JNK and p38 pathways generally participate in the response to oxidative stress and inflammation (66–68). A previous study showed that the RAS activator SHOC2 is a substrate of SCF^FBW7^. SCF^FBW7^ mediates the ubiquitination and proteolysis of SHOC2 after being phosphorylated by MAPK at Thr^507^ and blocks the RAS–MAPK pathway (69). CRL4^CRBN^ promotes the K48 linkage ubiquitination and the subsequent degradation of c-Jun and restrains the activity of the AP-1 complex, which leads to the down-expression of the pro-inflammatory factors inducible nitric oxide synthase (iNOS) and COX-2 (70). COP1, another Cul4-based E3 ubiquitin ligase, also regulates the protein stability of c-Jun. In mice, COP1 deficiency induced tumorigenesis and tumor progression depending on the upregulation of c-Jun (71). In addition, SCF^FBW7^ recognizes c-Jun after being phosphorylated by GSK3 and mediates the ubiquitination and the subsequent degradation of c-Jun by proteasome (72). CRL4^CRBN^ promotes a non-K48 linkage ubiquitination and degradation of the AMP-activated protein kinase alpha subunit (AMPKα). Notably, cereblon (CRBN) knockout decreases allergic responses in an AMPKα-dependent manner (73). CRBN plays an important role in the senescence process, and the depletion of CRBN activates p38/MAPK and downstream p53/p21 signaling and upregulates the senescence-associated markers SAHF (senescence-associated heterochromatic foci) and SA-β-Gal (74) (**Figure 1**, right).

### 3.3 JAK/STAT signaling pathway

The Janus kinase/signal transduction and activator of transcription (JAK/STAT) signaling pathway responds to a variety of inflammatory factors including cytokines, colony-stimulating factors, and growth factors. It also plays a central role in the pathogenesis of carcinoma and AIDs (75–77). Interleukins (ILs), interferons (IFNs), hormones, and colony-stimulating factors interact with specific type I/II cytokine receptors and induce the receptor dimerization and transphosphorylation of JAKs. As a step further, STATs are recruited and phosphorylated by JAKs, and the activated STATs dissociate from the receptors and form homodimers or heterodimers. The dimers then translocate into the nucleus and promote the transcription of associated genes (77, 78). The JAK family includes JAK1, JAK2, JAK3, and tyrosine kinase 2 (TYK2). Notably, in mammalian cells, STATs have seven subclasses: STAT1, STAT2, STAT3, STAT4, STAT5A, STAT5B, and STAT6 (78, 79). Targeting JAK signaling is thought to be a promising therapeutic strategy, and a series of JAK inhibitors (Jakinibs) have been approved by the US Food and Drug Administration (FDA) for the treatment of AIDs and lymphoma (76, 77).

CRL5^SOCS3^ promotes the ubiquitination and degradation of JAKs and STATs by the 26S proteasome (77, 80). The function of the negative regulation of the JAK/STAT signaling pathway makes suppressor of cytokine signaling (SOCS) proteins potential therapeutic targets for the treatment of JAK/STAT-associated diseases (81). Moreover, the Notch signaling pathway could transcriptionally activate Asb2, which is a SOCS-box-containing protein and is a SR of CRL5. Asb2 could replace SOCS in CRL5 and mediate the assembly of CRL5 and SCF^Skp2^ in a non-canonical E3 super complex, consequently promoting the degradation of JAK2 and E2A (82). SCF^HOS^ recognizes and interacts with the type I IFN receptor IFNAR1 in a phosphorylation-dependent manner upon the stimulation of IFN-α and subsequently promotes its degradation, which influences the function of the JAK/STAK signaling pathway in cells (83).

**Figure 2:**
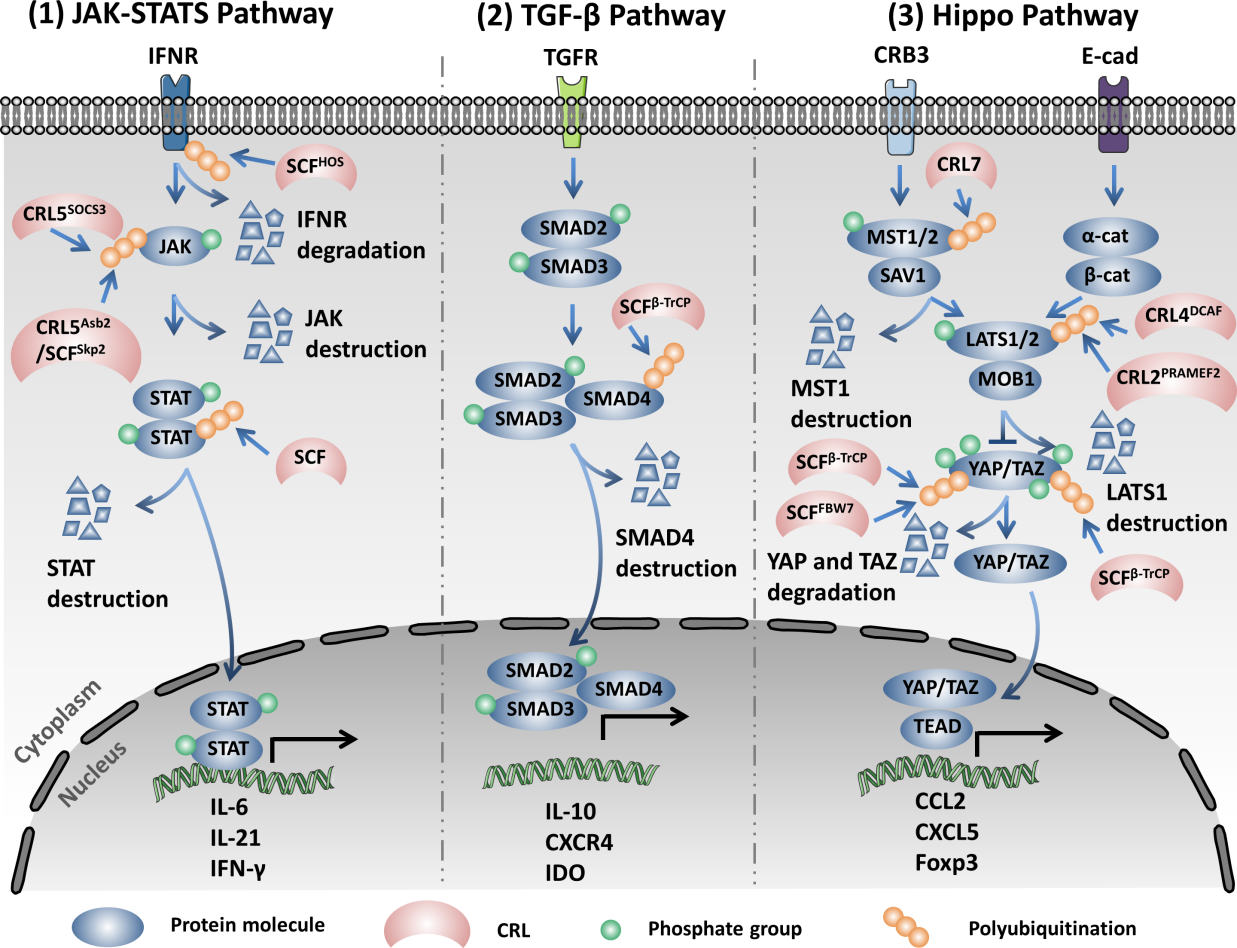
Cullin-RING ligases (CRLs) regulate the expression of multiple pro-inflammatory cytokines *via* modifying the Janus kinase/signal transduction and activator of transcription (JAK/STAT), TGF-β, and Hippo signaling pathways through the ubiquitination proteasome system (UPS). *Left*: SCF^HOS^ recognizes and interacts with the type I interferon (IFN) receptor IFNR1 in a phosphorylation-dependent manner upon stimulation of IFN-α, subsequently promoting the degradation of IFNR1. CRL5^SOCS3^ promotes the ubiquitination and degradation of JAK and STAT by the 26S proteasome. Asb2 mediates the assembly of CRL5 and SCF^Skp2^ in the non-canonical E3 super complex and promotes the degradation of JAK2. The HIV protein Vif (viral infectivity factor) mediates the host STAT1 and STAT3 ubiquitination and degradation *via* the Elongin–Cullin–SOCS-box binding motif. The Epstein–Barr virus (EBV) utilizes the host SCF (SKP1–Cul1–F-box) to promote the ubiquitination and degradation of STAT2 in the K48 linkage type. *Middle*: SCF^β-TrCP1^ specifically binds and destructs Smad4 to inhibit the expression of the genes in the TGF-β signaling pathway. *Right*: Cullin7 promotes the ubiquitination and destruction of Mst1. *PRAMEF2*, a substrate receptor (SR) of the Cul2-based ubiquitin ligase and CRL4^DCAF^ promotes the degradation of LATS1. SCF^β-TrCP^ and SCF^FBW7^ promote the degradation of the phosphorylated YAP through UPS. Moreover, SCF^β-TrCP^ mediates the ubiquitination and degradation of TAZ (transcriptional coactivator with PDZ-binding motif) in a phosphorylation-dependent manner.

The HIV virus protein Vif (viral infectivity factor) mediates the host STAT1 and STAT3 ubiquitination and degradation *via* the Elongin–Cullin–SOCS-box binding motif and subsequently reduces the production of the antiviral ISG15 induced by IFN-α (84). The Epstein–Barr virus (EBV) tegument protein BGLF2 utilizes the host SCF to promote the ubiquitination and degradation of STAT2 in the K48 linkage type by the 26S proteasome, which leads to the reduction of the expression of interferon-stimulating genes (ISGs) upon stimulation of IFN (85) (**Figure 2**, left).

### 3.4 TGF-β signaling pathway

Abnormal activation of the transforming growth factor beta (TGF-β) signaling pathway is one of the main causes of inflammatory diseases and cancers. TGF-β signaling plays a key role in remodeling the tumor microenvironment and in promoting immune tolerance and tumor evasion (86–88). The active TGF-β interacts with TGF-β type I and II receptors in the cell membrane. TGF-β type I and II receptors are activated through interacting with TGF-β, subsequently activating Smad2 and Smad3 *via* phosphorylation. The activated Smad2 and Smad3 then bind to Smad4 to form trimeric complexes and translocate into the nucleus to regulate the expression of associated genes (86, 87). SCF^β-TrCP1^ specifically binds to Smad4 instead of Smad2 and Smad3 to facilitate the ubiquitination and degradation of Smad4 and inhibits the expression of the genes in the TGF-β signaling pathway (89). Moreover, SCF^FBXL15^ promotes the ubiquitination and protein destruction of Smad ubiquitination regulatory factor 1 (Smurf1) by the 26S proteasome, which further regulates the bone morphogenetic protein (BMP) signaling pathway, thus affecting embryonic development and adult bone formation (90) (**Figure 2**, middle).

### 3.5 Hippo signaling pathway

The Hippo signaling pathway is an evolutionarily conserved pathway in mammalian cells that regulates a variety of biological processes including cell growth, tissue repair, organ regeneration, inflammation, and immunity. Dysregulation of the Hippo pathway leads to various human diseases such as cancer, AIDs, and abnormal development (91–93). The Hippo pathway is also involved in a series of kinase cascades in which MST1/2 interacts with SAV1 and phosphorylates SAV1, MOB1, and LATS1/2. Furthermore, the activated LATS1/2 subsequently mediates the phosphorylation of YAP/TAZ (yes-associated protein/transcriptional coactivator with PDZ-binding motif) at multiple sites and prevents them from translocating into the nucleus, ultimately inhibiting the transcription of the genes correlated with cell proliferation and survival. The activated Hippo signaling pathway plays a tumor suppressor role, while inactivated Hippo signaling promotes tumor progression (91, 93).

Overexpression of Cul4A is prevalent in human colon cancer (CC) cells. Cul4A induces the downregulation of MST1, LAST1, and p-YAP and promotes tumor progression by inactivating the Hippo pathway (94). Furthermore, a study on hepatocellular carcinoma (HCC) showed that the long non-coding RNA (lncRNA) uc.134 can inhibit the Cul4A-mediated ubiquitination and degradation of LATS1 and promote the phosphorylation of LATS1; moreover, it activates the Hippo pathway to suppress the cell proliferation of HCC (95). CRL4^Mahj^ promotes the ubiquitination and degradation of Wts, the ortholog of LATS1/2, therefore inactivating the Hippo pathway and contributing to the reactivation of neural stem cells (NSCs) in *Drosophila* (96). In other ways, PRAMEF2, a SR of Cul2-based ubiquitin ligases, promotes the degradation of LATS1 and subsequently induces the nuclear translocation of YAP. Subsequently, the nucleus-localized YAP transcriptionally activates the pro-proliferation genes to facilitate tumor progression (97). NEDD8-mediated NEDDylation of Cul7 promotes the ubiquitination and destruction of Mst1 and enables the translocation of YAP into the nucleus. Therefore, cell proliferation-related genes have been activated to promote cardiomyocyte proliferation and ventricular chamber maturation (98). Large tumor suppressor kinase (LATS) phosphorylates YAP at Ser^127^ and Ser^381^ and promotes the binding of YAP with 14-3-3 and cytoplasmic retention. The phosphorylated YAP will then recruit the E3 ubiquitin ligase SCF^β-TrCP^ and be destroyed in a proteasome-dependent manner. In addition, the NAE inhibitor MLN4924 could inhibit the activity of CRL4^DCAF^ to inhibit the degradation of LATS by CRL4^DCAF^, also promoting the phosphorylation and inactivation of YAP. MLN4924 in combination with the mammalian target of rapamycin–phosphatidylinositol-3-kinase (mTOR/PI3K) inhibitor GDC-0980 significantly suppresses the proliferation of *NF2*-mutant malignant pleural mesothelioma (MPM) cells (99). Moreover, previous studies indicated that FBW7 promoted the ubiquitination and destruction of YAP in HCC and *Kras* ^G12D^-driven pancreatic cancer. YAP silencing inhibited the tumorigenesis induced by *FBW7* depletion in *Kras* ^G12D^-dependent pancreatic cancer (100, 101). Therefore, both the cytoplasmic location and the degradation of YAP by UPS inhibit its oncogenic functions in the Hippo pathway (102). The apical polarity protein Crumbs (Crb) promotes the phosphorylation and the subsequent degradation of the Moesin domain protein Expanded (Ex) by SCF^Slimb/β-TrCP^, which inactivates the Hippo pathway through inhibiting the phosphorylation of the Hpo–Wts–Yki cascade (103). TAZ, another transcription coactivator, could also be phosphorylated by LATS, which primes it for further phosphorylation by CKI at the phosphorylation degron and recruits the SCF^β-TrCP^ E3 ligase for ubiquitination and degradation (104). Similarly, the PI3K/AKT signaling pathway modulates the protein abundance of TAZ through inhibiting the activity of GSK3, which catalyzes the phosphorylation of the N-terminal domain of TAZ and promotes its degradation by SCF^β-TrCP^ (105) (**Figure 2**, right).

### 3.6 Autophagy signaling pathway

Autophagy is a fundamental catalytic process of mammalian cells that contributes to the elimination of dysfunctional organelles, pathological proteins, and invading microbes upon stimulation by hypoxia, oxidative stress, and infection (106–108). Autophagy plays an important part in the pathogenesis of neurodegenerative disorders, AIDs, and carcinomas. The process of autophagy involves a series of autophagy-related proteins (ATGs), the autophagosome infusion with lysosomes, and the hydrolyzed cargos (106, 107, 109).

**Figure 3:**
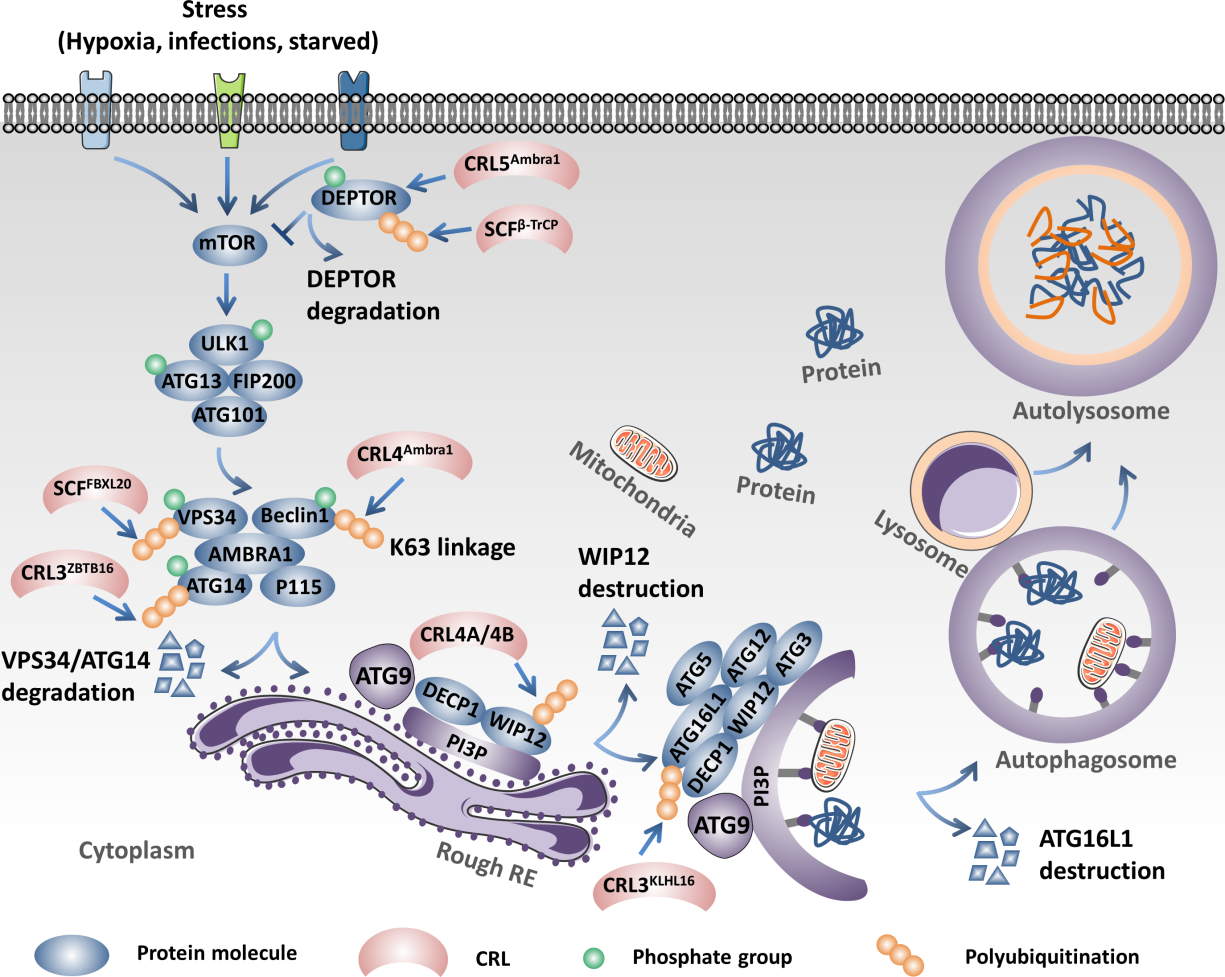
Cullin-RING ligases (CRLs) regulate the initiation and elongation of autophagy. Ambral1 binds with and inhibits Cul5, which leads to the accumulation of the mammalian target of rapamycin (mTOR) inhibitor DEPTOR, inactivating autophagy. However, SCF^β-TrCP^ mediates the ubiquitination and proteolysis of DEPTOR and activates autophagy. SCF^FBXL20^ facilitates the ubiquitination and degradation of VPS34 depending on the phosphorylation catalyzed by cyclin-dependent kinase (CDK). CRL4^Ambra1^ catalyzes the K63 linkage ubiquitination of Beclin1 under stimulation of starvation and promotes the interaction between Beclin1 and VPS34. CRL3^ZBTB16^ specifically promotes the ubiquitination and degradation of ATG14L. CRL4A- and CRL4B-based E3 ligases could induce the destruction of WIPI2 through the 26S proteasome to inhibit autophagosome biogenesis. CRL3^KLHL16^ promotes the K48 linkage ubiquitination and degradation of ATG16L1 and influences the elongation of the autophagosome.

CRL4^Ambra1^, as an E3 ligase, catalyzes the K63 linkage ubiquitination of Beclin1 under stimulation of starvation and promotes the interaction between Beclin1 and vacuolar protein sorting 34 (VPS34), which serves as a central component in the initiation of autophagy. However, WASH (Wiskott–Aldrich syndrome protein) competes with Ambra1 to bind to Beclin, reduces the K63 linkage of ubiquitination of Beclin1, induces the dissociation between Beclin1 and VPS34, and finally inhibits autophagy (110). In addition to being an E3 ligase, Ambra1, as a regulator, influences the activation and termination of autophagy by switching the interaction with Cul4 or Cul5. In detail, Cul4 binds with and reduces the abundance of the Ambral1 protein and inhibits the initiation of autophagy. Upon stimulation of autophagy, Ambral1 dissociates from Cul4 and binds with and inhibits Cul5, subsequently leading to the accumulation of the mTOR inhibitor DEPTOR and inactivating autophagy (111). However, SCF^β-TrCP^, another CRL, influences the initiation of autophagy through the negative regulation of the protein abundance of DEPTOR. SCF^β-TrCP^ mediates ubiquitination and the subsequent degradation of DEPTOR after phosphorylation in its conserved degron in an mTOR- and CK1-dependent manner (112–114). Furthermore, DNA damage triggers the activation of cyclin-dependent kinase (CDK). The activated CDK further promotes the phosphorylation of VPS34, which facilitates the ubiquitination and degradation of VPS34 by SCF^FBXL20^, finally inhibiting autophagy. Interestingly, DNA damage could also trigger the p53-mediated transcriptional activation of FBXL20 and regulate the initiation of autophagy (115). CRL3^ZBTB16^ could especially promote the ubiquitination and degradation of ATG14L, which plays a key role in the formation of the phagophore nucleation PI3KC3 complex I and promotes the initiation of autophagy. The antagonist of G-protein-coupled receptors (GPCRs) activates GSK3β, which decreases ZBTB16 and elevates the protein abundance of ATG14L, which then promotes autophagy and benefits treatment outcomes of neurodegeneration (116). CRL3^KLHL16^ promotes the K48 linkage ubiquitination and degradation of ATG16L and influences the elongation of the autophagosome (117). Moreover, both the CRL4A- and CRL4B-based E3 ligase could induce the destruction of WIPI2 through the 26S proteasome to inhibit autophagosome biogenesis during mitosis (118). SCF^FBXO27^, a glycoprotein-specific E3 ligase, regulates the ubiquitination of the lysosomal glycoproteins LAMP1/2, GSN, PSAP, and TMEM192 and the SNARE (SNAP receptor) proteins VAMP3 and VAMP7 upon lysosomal damage. After ubiquitin modification, the lysosomal proteins will recruit the autophagic machinery to launch lysophagy (119). The ubiquitination mediated by CRLs plays a central role in the onset and elongation of autophagy and influences selective autophagy, such as mitophagy and lysophagy (13, 120) (**Figure 3**).

### 3.7 Caspase signaling pathway

Caspases, members of the conserved cysteine protease family, play a critical roles in regulating cell apoptosis and inflammation (121, 122). The caspases involved in apoptosis signaling are caspases 3, 6, 7, 8, 9, and 10. Caspases 8, 9, and 10 are classified as initiator caspases, while caspases 3, 6, and 7 are executioner caspases. In humans, the caspases involved in inflammation are caspases 1, 3, 4, 5, and 12. Activation of the caspases in apoptosis or inflammation will induce programmed cell death or the release of inflammatory cytokines, including high-mobility group box (HMGB), IL-1β, and IL-18, which affect the development and progression of carcinomas and AIDs (121–123).

**Figure 4:**
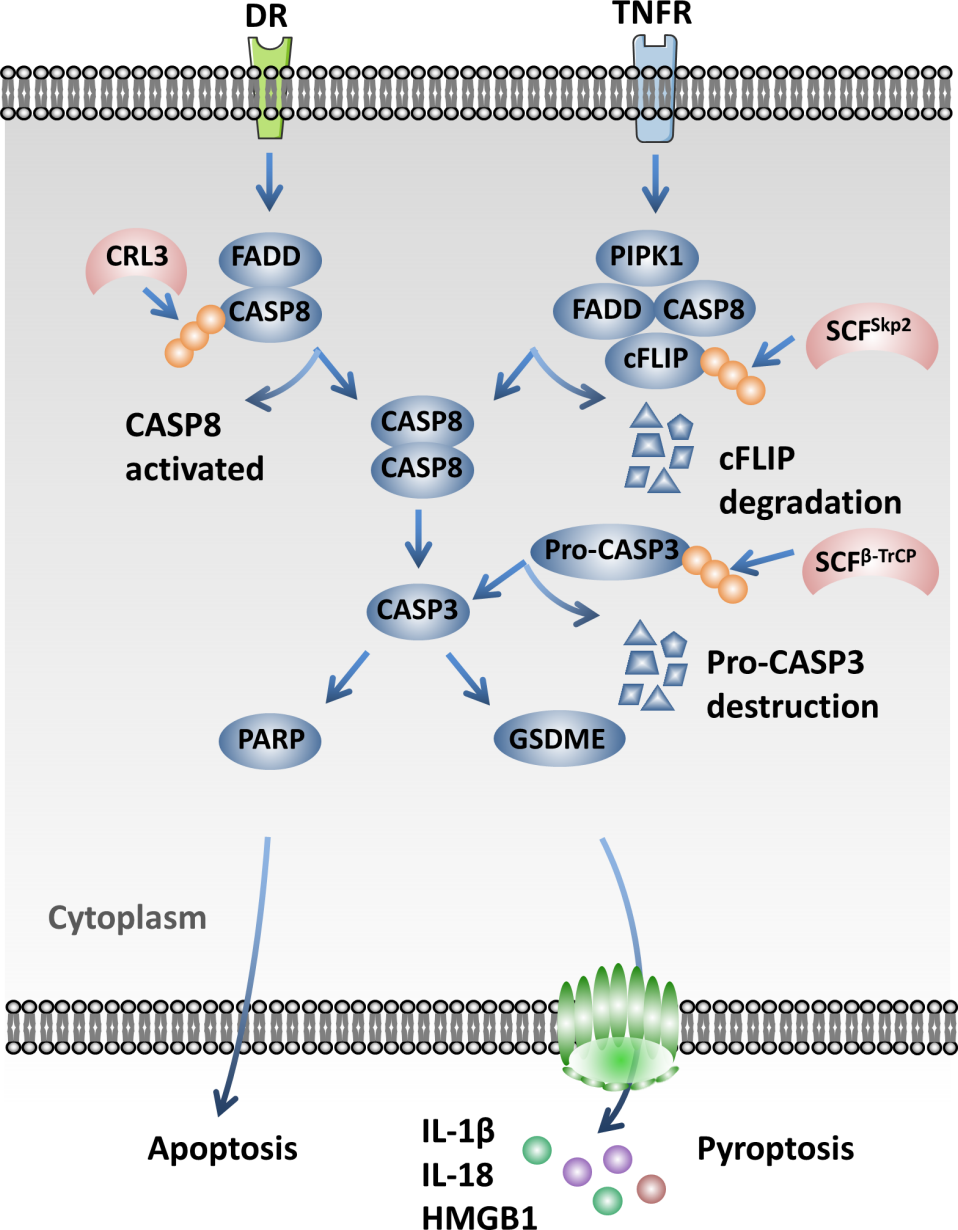
Cullin-RING ligases (CRLs) influence pyroptosis through regulating the caspase signaling pathway. Cul3-based ubiquitin ligases mediate the poly-ubiquitination and activation of caspase 8, which is essential for the assembly of the death-inducing signaling complex (DISC) under the treatment of extrinsic apoptosis signaling. SCF^Skp2^ promotes the ubiquitination and degradation of FLIP(L). SCF^β-TrCP^ mediates the degradation of pro-caspase 3 by the 26S proteasome and protects cells from apoptosis.

The NAE inhibitor MLN4924 inhibits the NEDDylation of Cullins and inactivates CRLs, resulting in the accumulation of activating transcription factor 4 (ATF4). ATF4 activates the transcription factor CHOP and then transcriptionally activates death receptor 5 (DR5) and caspase 8, which ultimately induces the extrinsic apoptosis of esophageal squamous cell carcinoma (ESCC) cells (124). It has been reported that the knockdown of CAND1 will activate caspase 8 and promote cell apoptosis in HCC *via* activating the CRLs (125). Interestingly, the Cul3-based ubiquitin ligase-mediated poly-ubiquitination and activation of caspase 8 is essential for the assembly of the death-inducing signaling complex (DISC) under the treatment of extrinsic apoptosis signaling. P62 associates with the DISC and promotes the aggregation of caspase 8 modified by Cul3 (126). SCF^Skp2^ promotes the ubiquitination and degradation of FLIP(L) and interrupts the interaction of p43-FLIP(L) and DISC. It also modulates the apoptosis mediated by TRAIL-R2 (DR5) (127). SCF^β-TrCP^ is the E3 ubiquitin ligase of pro-caspase 3 and mediates its degradation by the 26S proteasome, which protects cells from apoptosis (128) (**Figure 4**).

## 4 Cullin-RING ligases in autoimmune diseases

### 4.1 Systemic lupus erythematosus

Systemic lupus erythematosus (SLE) is termed based on the uncontrolled autoantibodies specific for the nuclear autoantigens, including double-strand DNA and the associated proteins induced to produce immune complex and tissue damage (129). SLE is a systemic AID that is triggered by genetic factors combined with a variety of environmental risk factors, such as exposure to ultraviolet radiation, smoking, infections, and environmental pollutants (129, 130). Autoreactive T and B cells produce diverse cytokines and autoantibodies that break the immune tolerance and induce immune dysfunction, promoting the occurrence of SLE. However, the pathological mechanisms of SLE remain largely unknown (129, 131).

Ubiquitination, as an important form of the PTM of proteins, regulates protein abundance, activity, subcellular localization, and their interactions, as well as a variety of signaling pathways. Ubiquitination plays a pivotal role in the pathogenesis of SLE (132, 133). A phase I clinical trial showed that the expression of Aiolos and Ikaros was significantly higher in patients with SLE than in healthy volunteers. CRLs, the largest family of E3 ubiquitin ligases, play a central role in the management of ubiquitin modification. CRBN, one of the SRs of CRL4, specifically promotes the transcriptional factors Ikaros (*IKZF1*) and Aiolos (*IKZF3*) for proteasomal degradation, which leads to the inactivation of T cells through downregulating the expression of IL-2. Lenalidomide and pomalidomide are immunomodulatory agents that activate T cells by promoting the ubiquitination and degradation of *IKZF1* mediated by CRL4^CRBN^ (134). Therefore, lenalidomide is used as an effective drug for the treatment of myeloma, which works by mediating the degradation of *IKZF1* and *IKZF3* in a CRL4^CRBN^-dependent manner (135).

Recent studies have shown that Iberdomide (CC-220), a new modulator of CRBN, interacts with CRBN at a higher affinity than lenalidomide or pomalidomide. It is used in the treatment of SLE. CC-220 promotes the binding of Aiolos and Ikaros to CRBN E3 ligase and the subsequent degradation by the 26S proteasome. CC-220 finally decreases the cell proliferation, plasmablast differentiation, and the immunoglobulin G (IgG) secretion of B cells stimulated by B-cell activating factor (BAFF) and CD40L, resulting in the attenuation of the progression of SLE (136, 137). CC-220 can help reduce the protein levels of Aiolos and Ikaros in B cells, T cells, and monocytes. In addition, CC-220 could markedly decrease the absolute population of CD19^+^ B cells and the expression of IL-1β and increase the production of IL-2 *ex vivo* (138). FBXW7 acts as a tumor suppressor by promoting the ubiquitination and degradation of various substrates including c-Myc, c-Jun, cyclin E, and MCL-1, which usually function as oncoproteins and promote tumor growth and survival (139, 140). SCF^FBXW7^ E3 ligase was also found to play a crucial role in the development of SLE. In tetramethylpentadecane (TMPD)-induced SLE, SCF^FBXW7^ induced cell apoptosis by promoting the K48 linkage ubiquitination and degradation of MCL-1. The apoptosis of peritoneal macrophages and neutrophils was lower in myeloid cell-specific *Fbxw7*-deficient (Lysm^+^Fbxw7^f/f^) C57BL/6 mice than in wild-type (WT) mice. In addition, the accumulation of immune complex, glomerulonephritis, the proliferation of glomerular mesangial cells, and the base membrane thickness decreased in the kidney of Lysm^+^Fbxw7^f/f^ mice. Fewer anti-Sm/RNP and anti-ANA autoantibodies and a reduced expression of major histocompatibility complex (MHC) II in B cells were found in Lysm^+^Fbxw7^f/f^ mice (141) (**Table 1**).

**Table 1:** Multiple functions of Cullin-RING ligases (CRLs) in autoimmune diseases (AIDs).

AID	CRL	Function	Pathogenesis	Modulator	Reference
SLE	CRL4^CRBN^	Attenuates the progression of SLE	CRL4^CRBN^ targets the transcriptional factors *IKZF1* and *IKZF3* for proteolysis by UPS, leading to the decrease of cell proliferation, plasmablast differentiation, and IgG secretion of B cells.	Lenalidomide Pomalidomide CC-220	(134–138)
	SCF^FBW7^	Accelerates the progression of SLE	SCF^FBW7^ induces cell apoptosis by degrading MCL-1. Lysm^+^FBxw7^f/f^ in SLE mice. It lowers apoptosis and decreases immune complex accumulation, glomerulonephritis, glomerular mesangial cell proliferation, and base membrane thickness in the kidney.	–	(141)
IBD	SCF^FBW7^	Promotes the progression of colitis	SCF^FBW7^ mediates the degradation of EZH2 and promotes the CX3CR1^int^ mononuclear phagocyte recruitment into colitis-affected colon tissues.	–	(26)
	SCF^FBW7^	Aggravates the intestinal inflammation of IBD	SCF^FBW7^ activates the NF-κB signaling pathway by degrading IκB and induces the aggravation of intestinal inflammation of IBD.	–	(25)
	SCF	Accelerates intestinal inflammation	Commensal bacteria generates ROS and butyrate to inhibit the neddylation of Cul1 and subsequently inhibits the NF-κB and β-catenin signaling pathways.	–	(142–146)
	SCF	Accelerates the severity of IBD	SCF degrades DEPTOR and activates mTOR, resulting in the acceleration of mucosal inflammation by enhancing the function of DCs.	MLN4924	(147)
	CRL2	Accelerates the inflammation of IBD	CRL2 mediates the degradation of HIF and accelerates the severity of IBD.	MLN4924	(148, 149)
	CRL3	Attenuates the severity of IBD	CRL3 promotes the degradation of Nrf2 and downregulates the expression of OGT, resulting in the inactivation of STAT3 and acceleration of IBD.	–	(150)
RA	SCF	Accelerates the progression of RA	SCF promotes IL-8 production, which increases the recruitment of inflammatory cells into the damaged joint area of RA.	–	(151)
	SCF	Accelerates the progression of RA	SCF promotes IκB degradation to activate the NF-κB pathway and promotes RA progression.	–	(152)
	SCF^FBXL19^	Attenuates the severity of RA	SCF^FBXL19^ promotes ST2 degradation and abrogates the pro-apoptotic and pro-inflammatory effects of IL-33 and relieves the symptoms of RA.	–	(153)
	CRL4^CRBN^	Attenuation the inflammation of RA	CRL4^CRBN^ promotes c-Jun degradation and inhibits the production of pro-inflammatory cytokines and attenuates the inflammation induced by LPS, relieving the severity symptoms of RA.	–	(70)
	CRL4B	Accelerates the severity of RA	CRL4B activates the Wnt pathway and increases the production of IL-1β and IL-8 to accelerate the severity of RA.	–	(154, 155)
Psoriasis	SCF	Promotes the progression of psoriasis	Cul1 is associated with the development of psoriasis. The mechanism remains unclear.	–	(156)
	CRL4^DACF2^	Attenuates the severity of psoriasis	CRL4^DACF2^ promotes NIK degradation and inhibits the non-canonical NF-κB pathway, leading to the reduction of the production of IL-23 and attenuating the severity of psoriasis.	–	(23)
T1DM	CRL3^KLHL3^	Accelerates the T1DM accompanied by hypertension	In *db*/*db* diabetes mouse, PKC phosphorylates KLHL3 on Ser433 and results in WNK4 accumulation, accelerating hypertension in T1DM.	–	(157, 158)
IPEX syndrome	CRL2^VHL^	Maintains the homeostasis and suppressive capacity of Tregs	The dysfunction of Tregs overactivates autoimmunity and leads to the occurrence of IPEX. VHL deficiency leads to the dysfunction of Tregs in a HIF-1α/IFN-γ/FOXP3-dependent manner.	–	(159)
	CRL	Maintains the homeostasis and suppressive function of Tregs	Treg-specific deletion of Rbx1, a catalytic subunit of CRL1–4, developed an early-onset fatal inflammatory disorder by disrupting the stability and suppressing the capacity of Tregs.	–	(160)

SLE, systemic lupus erythematosus; IBD, inflammatory bowel disease; RA, rheumatoid arthritis; T1DM, type 1 diabetes mellitus; IPEX syndrome, immune dysregulation, polyendocrinopathy, enteropathy, X-linked syndrome; UPS, ubiquitination proteasome system; ROS, reactive oxygen species; mTOR, mammalian target of rapamycin; DCs, dendritic cells: HIF, hypoxia-inducible factor; OGT, O-GlcNAc transferase; SCF, SKP1–Cul1–F-box; LPS, lipopolysaccharides; NIK, NF-κB-inducing kinase; PKC, protein kinase C; WNK4, kinase with-no-lysine 4; VHL, von Hippel-Lindau.

### 4.2 Inflammatory bowel disease

Crohn’s disease (CD) and ulcerative colitis (UC) are two forms of IBD that comprise a type of chronic and relapsing intestinal inflammation disease (161, 162). The pathological mechanism of IBD is complicated, which includes alterations of genomic and environmental risk factors, destruction of the gut microbiome barrier, and immune dysfunction (161–163).

PTMs such as phosphorylation, acetylation, and ubiquitination play critical roles in the pathogenesis and progression of IBD by modulating a variety of signaling pathways involved in its regulation (14, 164). CRLs, the largest family of E3 ubiquitin ligases, have been reported to regulate the development of IBD. SCF^FBW7^, one of the most important CRLs involved in the regulation of the inflammation pathway, is significantly correlated with the severity of IBD. Notably, SCF^FBW7^ promotes the progression of colitis through mediating the ubiquitination and degradation of the histone-lysine-*N*-methyltransferase enhancer of zeste homolog 2 (EZH2). The degradation of EZH2 results in the inhibition of H3K27me3 modification and increases the expression of CCL2 and CCL7 in CXCR1^hi^ macrophages, subsequently promoting the recruitment of CX3CR1^int^ pro-inflammatory mononuclear phagocytes (MPhs) into colitis-affected colon tissues. Myeloid deficiency of *FBW7* significantly alleviates the colitis induced either by dextran sodium sulfate (DSS) or 2,6,4-trinitrobenzene sulfonic acid (TNBS) in mouse models (26). On the other hand, SCF^FBW7^ activates the NF-κB signaling pathway by promoting the 26S proteasome-mediated IκB degradation and aggravates the intestinal inflammation in IBD. Interestingly, miR-129 could negatively regulate the expression of *FBW7* through promoting the 3′-UTR for degradation. Therefore, the upregulation of miR-129 reduces the inflammation of colitis induced by TNBS in a *FBW7*-dependent manner (25).

In addition, commensal bacteria influence the host intestinal homeostasis and play a pivotal role in regulating mucosal immunity and inflammation. A pioneer work by Neish et al. reported that prokaryotic microflora attenuated the inflammation of IECs by inhibiting the activation of the NF-κB signaling pathway through the blockage of the ubiquitination and degradation of IκBα (142). The group further found that commensal bacteria generated reactive oxygen species (ROS) and butyrate to inhibit the NEDDylation of Cul1 by inactivating the NEDD8-conjugating enzyme Ubc12, which led to the consequent blockage of the NF-κB and β-catenin signaling pathways (143, 144). The probiotic bacteria *Lactobacillus rhamnosus GG* (*LGG*) can induce the production of ROS and consequently inactivate Ubc12 and inhibit the NF-κB signaling pathway through blocking the NEDDylation of Cul1, which contributes to preventing necrotizing enterocolitis (NEC) and relieving IBD in neonates (145). Inhibition of NEDDylation modification could reduce mucosal inflammation and alleviate the severity of IBD in mice because inhibition of NEDDylation blocks the degradation of the mTOR inhibitor DEPTOR by the Cul1-associated CRL. The inactivation of mTOR subsequently inhibits the function of dendritic cells (DCs) and induces their apoptosis in an mTOR pathway-dependent manner (147).

Human umbilical cord mesenchymal stem cell-derived exosomes (hucMSC-exosome) contain high levels of miR-326 that could attenuate the NEDDylation of Cul1 and consequently inhibit the NF-κB signaling pathway, contributing to the relief of IBD induced by DSS in mice (146). Adrenomedullin (ADM) downregulates the inflammation of IECs due to the stabilization of HIF mediated by the deNEDDylation of Cul2 (148). In addition, pharmacological inhibition of NEDDylation by MLN4924 could stabilize HIF through the inhibition of Cul2 NEDDylation, which potentially attenuates IBD. Furthermore, human deNEDDylase-1 (DEN-1) could reduce the inflammatory response by promoting the deNEDDylation of Cullins (149).

The JAK/STAT signaling pathway plays a pivotal role in regulating inflammation. STAT3 has been reported to be closely correlated with the pathogenesis of IBD. Deficiency of the myeloid-derived STAT3 promotes the development of chronic enterocolitis through activating Th1 cells (165, 166). Similarly, deficiency of the IEC-specific STAT3 accelerates mucosal inflammation (167). Cul3-based E3 ubiquitin ligase promotes the proteolysis of nuclear factor erythroid 2-related factor 2 (Nrf2) and subsequently downregulates the expression of *O*-GlcNAc transferase (OGT), which is responsible for the *O*-GlcNAcylation of STAT3. Li et al. reported that the *O*-GlcNAcylation of STAT3 on T717 inhibited its phosphorylation and consequently accelerated the intestinal chronic inflammation in Cul3-deficient myeloid cells (150) (**Table 1**).

### 4.3 Rheumatoid arthritis

Rheumatoid arthritis (RA) is a chronic systemic AID. Joint pain and swelling are the prominent symptoms of RA. Anti-citrullinated protein antibodies (ACPAs), the RA-correlated autoantibody rheumatoid factor (RF), and C-reactive proteins (CRPs) are usually upregulated in patients with RA. The pathological mechanisms of RA include the change of the susceptibility genes and environmental risk factors. Of these, *HLA-DRB1* is the most important genetic risk factor, while smoking is the main environmental risk factor (168, 169).

Importantly, *Cul1* has been identified as one of the susceptibility genes of RA. *Cul1* is often highly expressed in T and B lymphocytes. Suppression of the expression of *Cul1* in T cells will reduce the production of IL-8. It has been reported that IL-8 plays a pivotal role in regulating the recruitment of inflammatory cells in the damaged joint area in RA (151). Another piece of research has also reported the close association of a promoter and two intronic polymorphisms of *Cul1* with RA and the methotrexate response in patients with RA (170). Bmi1 regulates the stability of IκBα through binding with the SCF E3 ubiquitin complex *via* its N-terminus after the phosphorylation by IKKα/β. Consistently, Bmi1 deficiency inhibits the NF-κB pathway *via* the accumulation of IκBα and attenuates arthritis (152).

Cytokines regulate the progression of RA through influencing the function of multiple immune cells, including T and B lymphocytes and mast cells. The IL-33/ST2 axis is closely correlated with the severity of RA. Pro-inflammatory cytokines are secreted by mast cells upon the stimulation of IL-33. Moreover, IL-33 could induce macrophages to produce chemokines and recruit neutrophils in RA-affected tissues. ST2 is the receptor of IL-33, and ST2 deficiency can relieve the symptoms of RA. IL-33 is highly correlated with the response of patients with RA to tumor necrosis factor (TNF) inhibitors (171, 172). SCF^FBXL19^ was found to promote the ubiquitination and degradation of ST2 by the 26S proteasome after phosphorylation on Ser442 by GSK3β and to abrogate the pro-apoptotic and pro-inflammatory effects of IL-33 (153).

CRL4^CRBN^ attenuates the inflammation induced by lipopolysaccharides (LPS) in a c-Jun-dependent manner and relieves the symptoms of inflammation-related diseases such as RA. CRL4^CRBN^ promotes the K48 linkage ubiquitination and degradation of c-Jun, therefore inhibiting the production of pro-inflammatory cytokines such as COX-2, iNOS, IL-1β, and IL-6 (70). Cul4B was significantly upregulated in the synovium and fibroblast-like synoviocytes (FLS) of adjuvant-induced arthritis (AIA) rats, which is a RA rat model. Cul4B promotes the activation of the canonical Wnt signaling pathway and the production of the pro-inflammatory cytokines IL-1β and IL-8, accelerating the severity of AIA. Therefore, MiR-101-3p plays an important role in anti-inflammation in AIA by reducing the expression of Cul4B (154). The elevated expression of circ_0015756 in FLS and the synovium of RA upregulates the expression of Cul4B by inhibiting the expression of miR-942-5p, consequently promoting the progression of RA by activating the canonical Wnt signaling pathway (155) (**Table 1**).

### 4.4 Psoriasis

Psoriasis is a prevalent chronic inflammatory skin disease worldwide. The pathogenesis of psoriasis is complicated. Genetic susceptibility, depression, smoking, obesity, and streptococcal infection could induce the occurrence and development of psoriasis.

IL-17 and IL-23 are the key inducers of psoriasis. Targeting IL-17, IL-23, and TNF-α has been considered as the predominant therapeutic strategy for the treatment of psoriasis (173, 174). A study based on the microarray data of a cDNA library indicated that Cul1 is highly associated with the development of psoriasis (156). CRL4^DCAF2^ negatively regulates the production of IL-23 through the ubiquitination and degradation of NIK, which is associated with the non-canonical NF-κB pathway. Therefore, DCAF2 deficiency induced the accumulation of NIK, promoted the activation of the NF-κB non-canonical signaling pathway, and increased the production of IL-23. In a mouse model, MLN4924 treatment accelerated the severity of psoriasis through the inactivation of CRLs, including CRL4^DCAF2^. Furthermore, DCAF2 DC-conditional knockout mice showed increased susceptibility to AIDs (23) (**Table 1**).

### 4.5 Type 1 diabetes

Type 1 diabetes mellitus (T1DM) is IDDM and is an organ-specific AID and a severe metabolic disease. T1DM is frequently accompanied by the occurrence of hypertension. Autoreactive T cells and other components of the immune system attack pancreatic B cells to induce the occurrence of T1DM. Currently, multiple susceptibility genes and environment risk factors associated with the development of T1DM have been identified. Human leukocyte antigen (*HLA*) is one of the major susceptibility genes related to the autoantigen recognition and immune tolerance of T cells. Infections, commensal bacteria, and diet are the important environmental risk factors that influence the development of T1DM by modulating the functions of the immune system (175, 176).

Cul3^KLHL3^ is a SR of E3 ubiquitin ligase and plays an essential role in hypertension. Kinase with-no-lysine 4 (WNK4), a bona fide substrate of KLHL3, modulates the activation of the Na–Cl cotransporter (NCC). The phosphorylation of KLHL3 on Ser433 of the Kelch domain by protein kinase C (PKC) inhibited the interaction of WNK4 and KLHL3 (177). The phosphorylation of KLHL3 by PKC was also observed in the kidney of *db*/*db* mice (157). In streptozotocin-induced T1DM, the expression of KLHL3 was significantly decreased, therefore inducing the activation of the WNK–NCC cascade. Furthermore, KLHL3 plays a pivotal role in renal sodium reabsorption in conditions of T1DM (158) (**Table 1**).

### 4.6 Immune dysregulation, polyendocrinopathy, enteropathy, and X-linked syndrome

Immune dysregulation, polyendocrinopathy, enteropathy, X-linked (IPEX) syndrome is a multisystem AID with diverse clinical syndromes including enteropathy, skin manifestations, and endocrinopathy. Loss-of-function mutations in the transcription factor forkhead box P3 (*FOXP3*) are the dominant factors that induce the occurrence of IPEX (178–180). *FOXP3* is mainly expressed on CD4^+^CD25^+^ regulatory T cells (Tregs) and modulates their development and functions. *FOXP3* deficiency leads to the downregulation of a few of the core signature genes in Tregs and suppresses the activity of these cells. The dysfunction of Tregs overactivates autoimmunity and leads to the occurrence of IPEX (181).

A previous study indicated that the von Hippel-Lindau (VHL) E3 ubiquitin ligase played a pivotal role in regulating the functions of Tregs. VHL interacts with Elongins B and C, Cul2, and Rbx1 to form the CRL2 complex, which mediates the ubiquitination and subsequent degradation of HIF-1α. Conditional knockout of VHL in Tregs leads to the accumulation of HIF-1α, which induced the production of IFN-γ. VHL deficiency is accompanied with the downregulation of *FOXP3*, which can be reversed by IFN-γ deprivation. In conclusion, VHL modulates the stability and immunosuppressive functions of Tregs in a HIF-1α/IFN-γ/FOXP3-dependent manner (159). In addition, a recent study has suggested that Tregs with depletion of Rbx1, a catalytic subunit of CRL1–4, developed an early-onset fatal inflammatory disorder due to their disrupted stability and suppressive capacity. Moreover, deficiency of the Ube2m, but not the Ube2f, NEDDylation conjugation enzyme in Tregs presented similar but less severe phenotypes compared to Rbx1 deletion. Therefore, the Ube2m–Rbx1 axis plays a crucial role in regulating the homeostasis and function of Tregs (160) (**Table 1**).

## 5 Conclusion and outlook

AIDs are chronic inflammation-associated diseases induced by the dysfunction of the immune system. Overactivated autoreactive immune cells such as T cells, DCs, and macrophages produce pro-inflammatory cytokines, including IL-1β, IL-6, IL-17, IL-23, COX-2, and iNOS, or chemokines that promote the recruitment of inflammation-associated cells in the affected areas of AIDs. Ultimately, the tissue injury and the severity of AIDs are further accelerated (5). Hyper-activation of the inflammation-associated signaling pathways such as NF-κB, JAK/STAT, MAPK, and TGF-β is prevalent during the occurrence and progression of AIDs, including IBD, SA, and SLE.

Given that the activation of the inflammation-associated signaling pathways plays critical roles in the initiation and progression of AIDs, it is important to explore the underlying mechanisms of the abnormal activation of the key genes related to AIDs. These mechanisms include the transcriptional regulation of genes and the PTM of the correlated proteins. Ubiquitination, one of the PTMs of proteins, plays a pivotal role in regulating their stability, activation, and localization. The abnormal regulation of ubiquitination is closely related to the activation of the genes in the inflammatory pathways and the progression and recurrence of AIDs (10). CRLs comprise the largest class of E3 ubiquitin ligases and include more than 300 members that regulate the stability of about 20% proteins in a proteasome-dependent manner in mammalian cells (50). The results from clinical and animal model studies revealed that the dysregulation of CRLs usually functions as an inducer or an inhibitor in the development of inflammation and in the progression of AIDs. For instance, SCF^FBW7^ functions as a regulator of AIDs in the following aspects: 1) promoting the degradation of IκB, EZH2, and MCL-1; 2) accelerating the pro-inflammatory NF-κB signaling pathway; 3) promoting the recruitment of MPhs into colitis-affected colon tissues; 4) increasing the apoptosis of macrophages and neutrophils; and 5) leading to the accumulation of immune complex and inducing the aggravation of SLE (25, 26, 141). On the other hand, CRL4^CRBN^ promotes the degradation of IKZF1, IKZF3, or c-Jun, which leads to the reduction of the secretion of IgG or the pro-inflammatory cytokines COX-2, iNOS, IL-1β, and IL-6, consequently attenuating SLE or RA (70, 138) (**Table 1**).

The development of small molecules targeting CRLs will be a promising therapeutic approach in the clinical intervention of AIDs. In line with this notion, treatment with the NEDDylation inhibitor MLN4924 effectively decreases inflammation through inhibiting the activation of SCF E3 ligases in IBD (143, 144, 147). Immunomodulatory imide drugs (IMiDs) such as thalidomide and its derivatives lenalidomide, pomalidomide, and Iberdomide (CC-220) act as molecular glue degraders to promote the ubiquitination and degradation of IKZF1 and IKZF3 by CRL4^CRBN^, ultimately relieving the symptoms of SLE (134, 137, 138, 182) (**Table 1**). Proteolysis-targeting chimera (PROTAC) technology is another novel targeted protein degradation (TPD) method that has rapidly developed in recent years. As bifunctional small molecules, PROTACs induce the ubiquitination and proteolysis of target proteins by E3 ubiquitin ligases (183). In addition, small molecules targeting the interface between CAND1 and Cullins comprise a new pharmocological strategy based on UPS. A recent study has shown that the chemical probe C60 perturbs the normal interaction between CAND1 and Cul1, resulting in the accumulation of p53, thus inducing the reactivation of EBV from latency (184). Either small-molecule inhibitors or the emerging TPD technology involving molecular glue degraders and PROTACs could be effective thrapeutic strategies to benefit the intervention of inflammation and AIDs. In particular, the TPD method, a novel pharmacological strategy to degrade the protein of interest (POI) using small-molecule degraders *via* hijacking CRLs, could be promising for the treatment of AIDs in the near future.

## Author contributions

XYZ and XD conceived the manuscript. XYZ wrote the manuscript with partial help from YL, TZ, and YT. XLZ, Y-GY, and XD edited and revised the manuscript. All authors contributed to the article and approved the submitted version.
